# Predictive Utility of Cerebral Blood Flow Transients in Experimental Stroke

**DOI:** 10.1007/s12975-025-01410-9

**Published:** 2026-01-22

**Authors:** Janos Lückl, Monika Szücs, Ferenc Rarosi, Amirhossein Salehzadeh, Jens P. Dreier

**Affiliations:** 1 Center for Stroke Research Berlin, Charité – Universitätsmedizin Berlin, corporate member of Freie Universität Berlin, Humboldt-Universität zu Berlin, Berlin Institute of Health, Berlin, Germany; 2 Department of Neurology, RWTH Aachen University, Aachen, Germany; 3 Department of Medical Physics and Informatics, University of Szeged, Szeged, Hungary; 4 Department of Experimental Neurology, Charité – Universitätsmedizin Berlin, corporate member of Freie Universität Berlin, Humboldt-Universität zu Berlin, Berlin Institute of Health, Berlin, Germany; 5 Department of Neurology, Charité – Universitätsmedizin Berlin, corporate member of Freie Universität Berlin, Humboldt-Universität zu Berlin, Berlin Institute of Health, Berlin, Germany; 6 Bernstein Center for Computational Neuroscience Berlin, Berlin, Germany; 7 Einstein Center for Neurosciences Berlin Department of Neurology, Charité, Universitätsmedizin, Berlin, Germany

**Keywords:** Risk stratification, Filament occlusion, Spreading depolarization, The slope of flow transients

## Abstract

A gap in developing novel preclinical treatment strategies for ischemic stroke is predicting long-term outcome in experimental stroke models early during ischemia to reduce heterogeneity and sample size. Besides saving costs through improved risk stratification, reducing the number of animals is a requirement of the 3Rs principle. In this secondary analysis, we analyzed 28 Sprague-Dawley rats of a prospective data base that underwent 90-minute filament-occlusion of the middle cerebral artery (MCAO) to assess the predictive power of early variables at 30, 60, and 90 min after occlusion. The animals were sacrificed after 72 h. Infarct sizes were determined by hematoxylin staining. In a minimally invasive fashion, we recorded cerebral blood flow (CBF) with laser-Doppler flowmetry and direct current (DC)/alternating current-electrocorticography (ECoG) with epidural Ag/AgCl electrodes. Both CBF and ECoG markers correlated with the cortical infarct volumes. Multiclass receiver operating characteristic analysis identified the best predictors of three risk classes, and Spearman’s rank correlation was used to explore relationships between ECoG and CBF. The slope of the CBF transients in response to spreading depolarization (SD) was the best biomarker at all time points, while the DC integral was the best epidural biomarker. Both correlated negatively at all time points (ρ < -0.68). In summary, we have found that early risk stratification during MCAO in rats is possible using minimally invasive biomarkers. This would enable, in particular, the early sorting out of animals with a low risk of cortical infarction in neuroprotection studies, where these animals typically distort the statistical results.

## Introduction

Stroke is the second leading cause of death and third leading cause of disability worldwide [1]. Just a few decades ago, acute ischemic stroke was an untreatable condition. Historically, the introduction of stroke units and the treatment of vascular risk factors were the first steps towards improving patient outcomes [2]. Building on this, the randomized controlled trials of endovascular treatment in addition to intravenous thrombolysis have recently led to a breakthrough in effective treatment in selected cases. Thus, fewer than three patients need to be treated to achieve a beneficial change in outcome when a major artery of the anterior circulation is occluded and recanalization is initiated within six hours [3, 4]. However, similarly successful therapeutic strategies are lacking for many other forms of ischemic stroke, and treatment can probably be further optimized even for patients who are eligible for recanalization therapies.

The development of intravenous thrombolysis with recombinant tissue plasminogen activator (rtPA) as a rescue therapy for ischemic stroke was originally based on experiments on rabbits [5]. Unfortunately, the translational development of other pharmacological strategies for neuroprotection based on preclinical studies has so far failed [6, 7]. Nevertheless, animal experiments have contributed significantly to a better understanding of the pathophysiological changes in the parenchyma that precede the evolution of neuronal damage during cerebral ischemia. The modern concept focuses on spreading depolarization (SD) with a terminal increase in extracellular K^+^ concentration ([K^+^]_o_) and a terminal negative direct current (DC) shift in zones with severe ischemia, which spreads slowly as a wave and becomes progressively shorter in tissue zones with increasingly adequate perfusion and supply of oxidative substrates [8–11]. On this basis, Astrup and colleagues developed the concept of the ischemic core and penumbra in 1977 using animal experiments with K^+^-selective microelectrodes [12, 13]. Accordingly, cerebral ischemia involves a dual threshold with a core area in which cerebral blood flow (CBF) is so low that terminal SD occurs, and a penumbra in which tissue initially recovers spontaneously from SD but brain activity is persistently suppressed, as evidenced by reduced amplitudes of brain activity in AC-electrocorticography (ECoG) recordings. It is important to note that, for example, after occlusion of the middle cerebral artery (MCAO) in rats, neuronal death only begins when SD persists in at least one location for longer than 15 min [14]. If CBF is restored within this time window, SD is reversible and no neuronal death is observed histologically 72 h later, despite severe ischemia and persistent SD during the ischemic phase. Recent clinical studies have largely confirmed the crucial and at the same time complex role of SDs in ischemic strokes of various etiologies in patients [14–18]. For example, consistent with the experimental hypothesis that SD initiate not only the conversion of the ischemic core to dead tissue but also the conversion of the ischemic penumbra to dead tissue [19–21], cumulative SD-induced depression duration was recently shown to correlate positively with late infarct progression in patients with malignant hemispheric stroke (MHS) [17].

Recognizing that SD and related processes—such as depression of spontaneous activity or hemodynamic responses—occur in both animals and patients could also help create new strategies to improve the design of preclinical animal studies. Thus, focal cerebral ischemia is clinically characterized by a large heterogeneity in terms of outcome [22, 23], a finding that is also strongly reflected in animal studies [24, 25]. For example, our previous animal studies on MCAO in rats indicated in particular that cortical infarct size exhibits notable variability, even with strict quality control during experiments [26]. Some animals do not even develop a cortical infarct at all, or the infarct size is negligible. The heterogeneity of infarct size likely results from multiple factors, including individual variations in the anatomy of the circle of Willis and other collateral structures, variations in the coagulation system and/or platelets, endogenous disposition to SD, hemodynamic responses to SD and other modifiers of vascular tone, stalled neutrophils and monocytes/macrophages trapped in the capillary network, and the choice of the anesthetic, just to name a few [8, 24, 26–31].

Here, we hypothesize that the use of SD-related biomarkers during MCAO for reliable early prediction of infarct size could improve the statistical power of studies on neuroprotective drugs. Then, a given neuroprotective intervention could be randomized during the experiment according to different risk classes. An important prerequisite for a suitable biomarker would be that it should be easy to collect, i.e. minimally invasive. To this end, this is a secondary analysis of our previous prospective study to characterize SD in the MCA territory of Sprague-Dawley rats after MCAO [14]. Specifically, we analyzed the 28 animals in group 1 A with 90-minute MCAO in which (i) two epidural electrodes recorded DC/AC-ECoG and (ii) a laser-Doppler flowmetry (LDF) probe recorded CBF. Our research question was to compare the different early biomarkers that emerged from the recordings and to determine the biomarker that best discriminated between three predefined risk classes based on the cortical infarct volumes at sacrifice of the animals 72 h after MCAO.

## Methods

The reporting complies with the ARRIVE Guidelines. The experiments were previously described in detail [14]. In brief, all animal experiments were authorized by the animal welfare authorities in Berlin, Germany: Berlin State Office for Health and Social Affairs (LAGeSo), G0152/11, and all experimental procedures were conducted in accordance with the Charité Animal Welfare Guidelines. The animals were housed in groups (two animals/cage) under a 12-hour light/dark cycle with food and tap water available ad libitum. Naive, male, wild-type Sprague-Dawley rats (*n* = 28; 300–340 g; Charles River) were anesthetized with isoflurane 4% for induction and 1.2%–1.6% for maintenance in a mixture of 70% N_2_O and 30% O_2_. Body temperature was maintained at 37.5 ± 0.5 °C using a homoeothermic control unit (Homeothermic Blanket Control, Harvard Apparatus, Cambridge, MA, U.S.A.). A polyethylene catheter (PE-50) was inserted into the tail artery to monitor systemic arterial pressure (Pressure Monitor BP-1, World Precision Instruments, Berlin, Germany). In addition, arterial partial pressure of O_2_ (pO_2_,), pCO_2_, and pH were serially controlled using a Compact 1 Blood Gas Analyzer (AVL Medizintechnik, Graz, Austria). Adequacy of the level of anesthesia was assessed by testing motor and blood pressure responses to tail pinch.

**Fig. 1:**
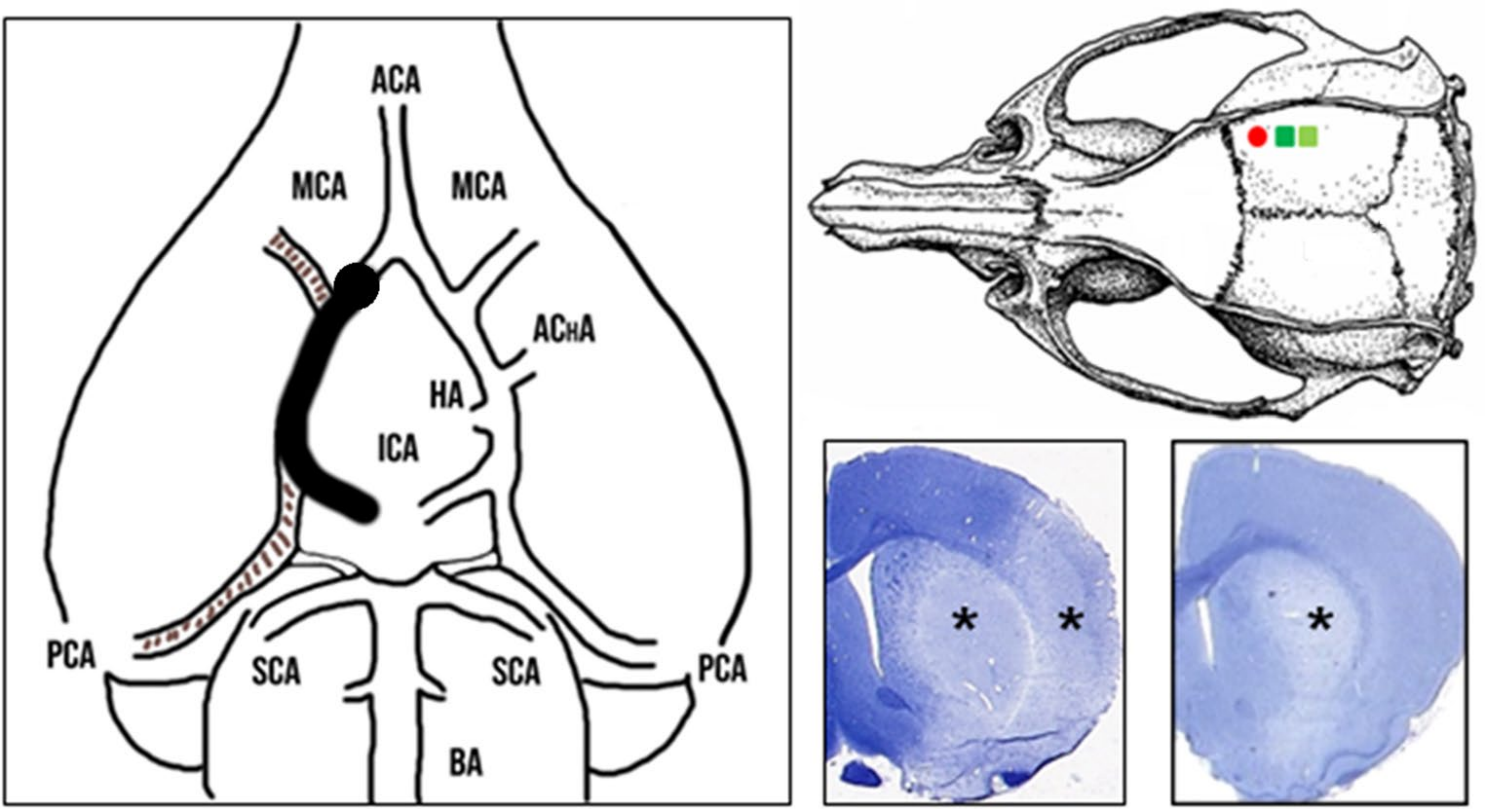
The intraluminal filament occlusion of the middle cerebral artery (MCA) is illustrated in the left panel. A silicone-coated nylon filament was inserted through the common carotid artery into the internal carotid artery and advanced until laser-Doppler flowmetry (LDF) indicated sufficient MCA occlusion (MCAO). The upper right panel displays the experimental setup, with the recording site of the LDF probe (red, filled circle) and the positions of the two epidural Ag/AgCl electrodes (green squares). The lower right panel shows hematoxylin-stained sections from two different animals. The left image depicts an ischemic infarct affecting both the striatum and the cortex (asterisks), while the ischemic infarct in the right image only affects the striatum (asterisk) but not the cortex. ACA = anterior cerebral artery; AChA = anterior choroidal artery; BA = basilar artery; CCA = common carotid artery; HA = hypothalamic artery; ICA = internal carotid artery; PCA = posterior cerebral artery; SCA = superior cerebellar artery

Head surgery and filament occlusion of the MCA were performed as explained previously [14]. The LDF probe (tip diameter: 1 mm, fiber separation: 0.25 mm) attached to a flowmeter (PeriFlux 4001, Perimed AB, Järfälla, Sweden) was fixed to the skull with dental cement (Paladur, Heraeus Kulzer GmbH, Hanau, Germany) over a burr hole with a diameter of 1 mm (4–5 mm lateral to midline, 1–2 mm posterior to bregma) (Fig. 1). The epidural DC/AC-ECoG was measured with two Ag/AgCl electrodes fixed with dental cement over the territory of the right MCA (4–5 mm lateral to midline, ~ 4 mm and ~ 5 mm posterior to Bregma) connected to a differential amplifier (Jens Meyer, Munich, Germany). The reference electrode was implanted under the skin in the nuchal region. Analog-to-digital conversion was achieved using a Power 1401 (Cambridge Electronic Design Limited, Cambridge, UK). Mean arterial pressure (MAP), DC/AC-ECoG and CBF were continuously recorded using a personal computer and Spike 2 software (Cambridge Electronic Design Limited, Cambridge, UK).

After 72 h of survival, all animals underwent cardiac perfusion fixation with saline and 4% paraformaldehyde (Sigma-Aldrich, St. Louis, MO, USA) as described previously [14]. In brief, 20 μm coronal cryosections of the brains were serially collected at 1 mm intervals. Sections were stained with hematoxylin according to the standard protocol of the Department of Experimental Neurology of the Charité – Universitätsmedizin Berlin. The stained sections were scanned (600 dpi) to determine the size of the infarction using a computer-based image analyzer (Sigma Scan Pro 5.0, Systat, San Jose, USA). Lesion volumes in cortex and striatum were determined by summation of the infarct areas of 10 brain slices integrated by the thickness. In addition, we measured the left and right hemisphere volumes to apply an edema correction [32].

### Data Analysis

The analysis of electrophysiological and CBF-related parameters was conducted using LabChart data analysis software (ADInstruments, Dunedin, New Zealand). To streamline workflow, we created mini-windows to calculate the mean of a given interval, as well as the amplitude and duration of CBF transients. The analysis of the raw data was performed by A.S. in a blinded manner and supervised by J.L.

**Fig. 2:**
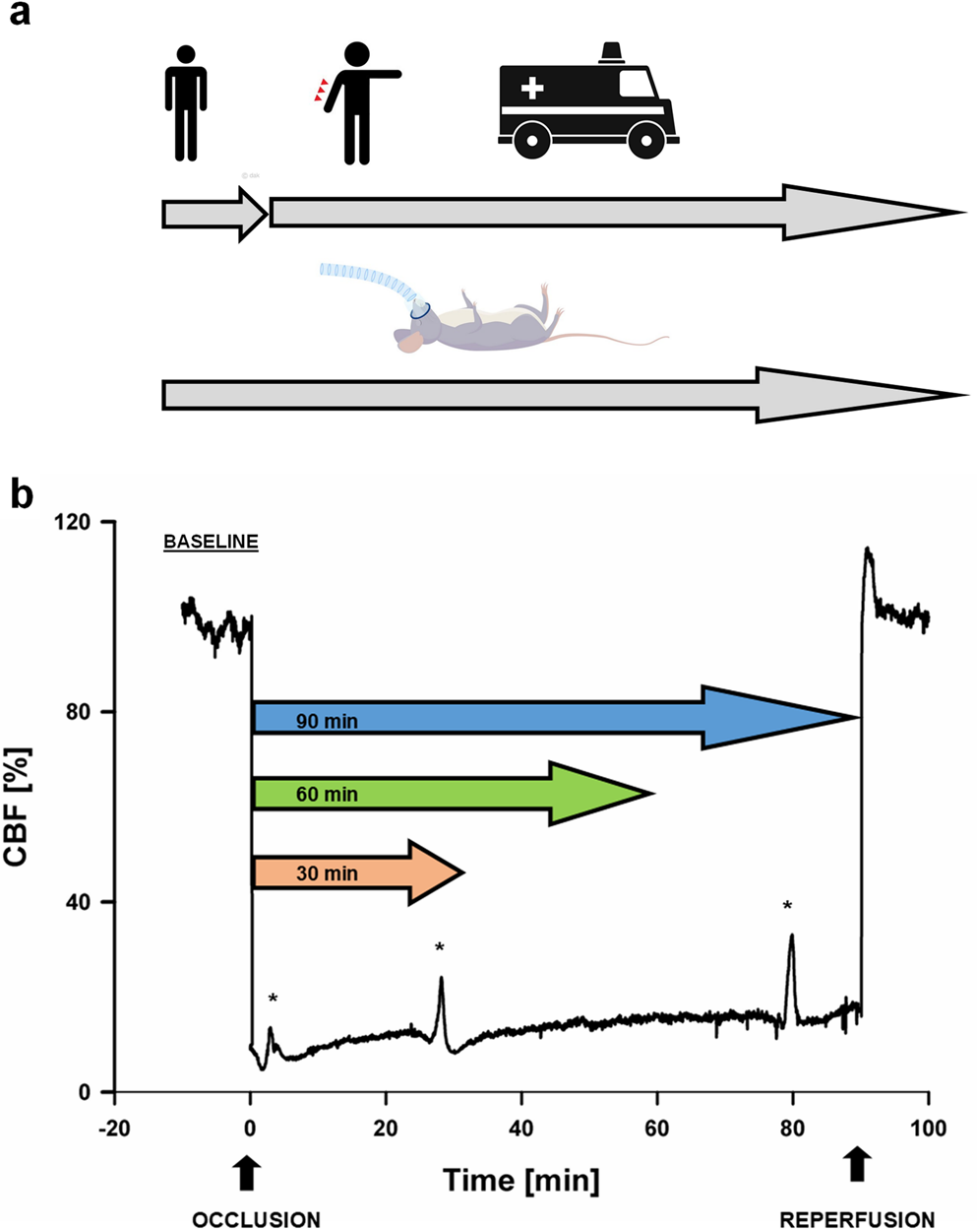
The figure presents the basic concept and the design of the study. (**a**) shows the different approaches to risk assessments between humans and experimental animals. A neurological examination can be carried out in patients at any time. Since the neurological status of an anesthetized animal cannot be assessed, risk stratification can only be performed in animal studies with the help of biomarkers. (**b**) summarizes the design of the analysis. The average flow (MEAN-CBF) during 30, 60, and 90 min of ischemia as a fraction of baseline flow as well as the number (NUM-FT) and different parameters (amplitudes, slopes) of the flow transients (designated with asterisks) were calculated

**Fig. 3:**
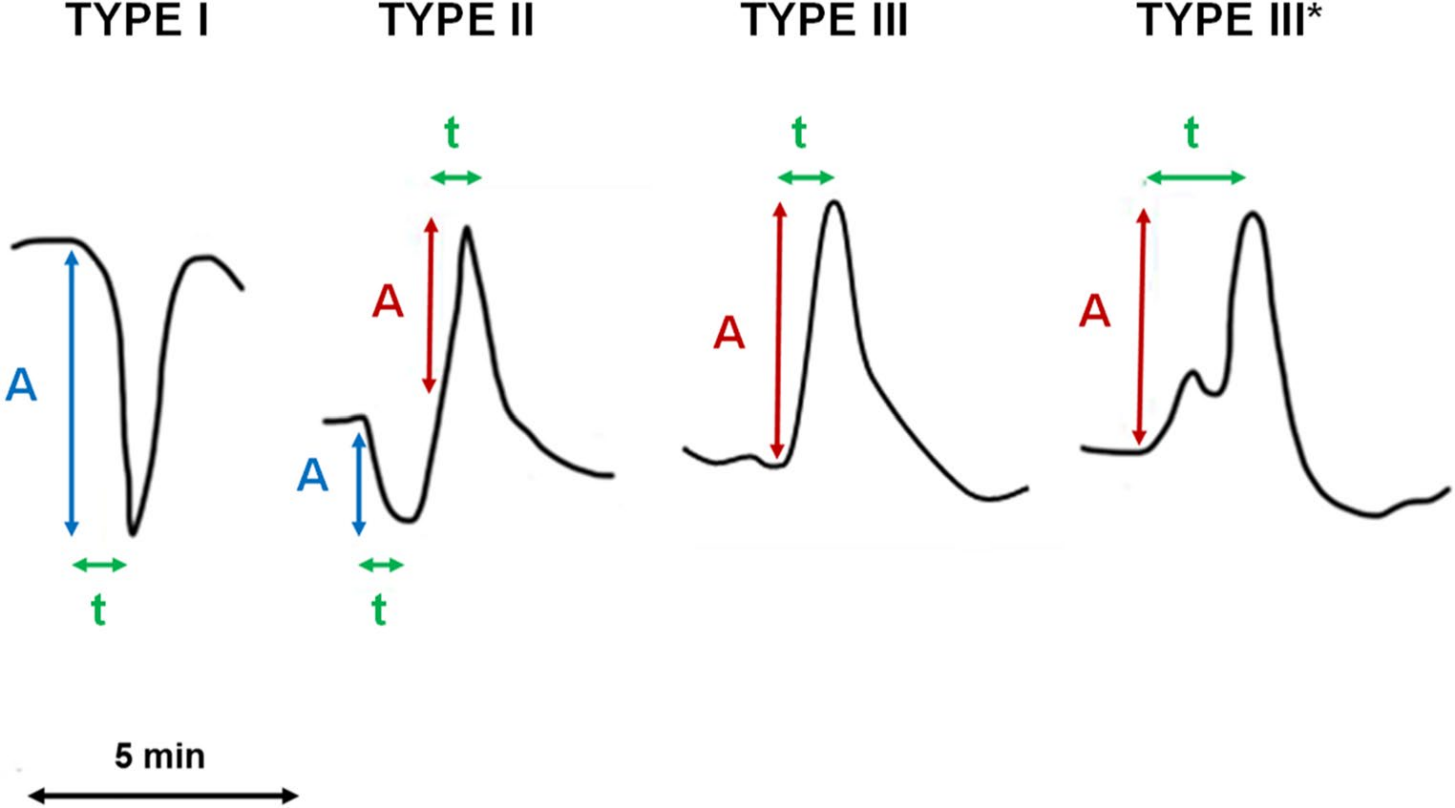
The flow transients were subsequently classified by their morphology into three main types. Type I transients were characterized by a negative, monophasic (hypoemic) waveform. Type II transients exhibited a biphasic waveform that included both hypo- and hyperemic components. Type III transients were characterized by a positive, monophasic (hyperemic) waveform. Occasionally, a single dip (Type III*) could be observed in the morphology of the transient. Both the negative (N-AMP, blue vertical lines) and the positive components (P-AMP, red vertical lines) of the amplitudes were measured separately. In cases where a positive amplitude was interrupted by a turn (Type III*), we always considered the higher amplitude. The slopes of the transients were calculated as the amplitude divided by the time (green horizontal lines) it took CBF to reach its positive (P-SLOPE) or negative (N-SLOPE) peak level. A = amplitude, t = time

The average residual flow (MEAN-CBF) during 30, 60, and 90 min of ischemia, as well as the CBF during reperfusion, and the amplitude of CBF transients were calculated relative to baseline CBF (= 100%) prior to MCA occlusion (Figs. 2 and 3). SD-induced CBF transients were observed as a CBF change of more than 3% with a duration of more than 60 s, accompanied by an SD-typical negative DC shift and without MAP fluctuation that could have explained the CBF change [26]. The CBF transients were subsequently classified by their morphology into three distinct types (Fig. 3). Type I transients were characterized by a negative, monophasic (hypoemic) waveform. Type II transients exhibited a biphasic waveform that included both hypo- and hyperemic components. Type III transients were characterized by a positive, monophasic (hyperemic) waveform. Occasionally, a single dip (Type III*) could be observed in the morphology of the transient. Both negative (N-AMP) and positive components (P-AMP) of the amplitudes were measured separately. In cases where a positive amplitude was interrupted by a dip (Type III*, *n* = 7), we always considered the higher amplitude. Finally, the total peak-to-peak amplitude (T-AMP) was determined as T-AMP = P-AMP + N-AMP. The positive slope of a given transient (P-SLOPE) was defined as P-AMP divided by the time it took CBF to reach the positive peak from its starting point. Accordingly, the negative slope of a given transient (N-SLOPE) was defined as N-AMP divided by the time it took CBF to reach the negative peak from its starting point. In addition to MEAN-CBF, the number of the CBF transients (NUM-FT) and the mean values of the parameters (amplitudes, slopes) were calculated for three time intervals: 0–30 min, 0–60 min, and 0–90 min following the onset of ischemia (Fig. 2). We also introduced the parameter ALL-SLOPES, which represents the average value of all positive and negative slopes found within the given time intervals (30, 60 and 90 min).

**Fig. 4:**
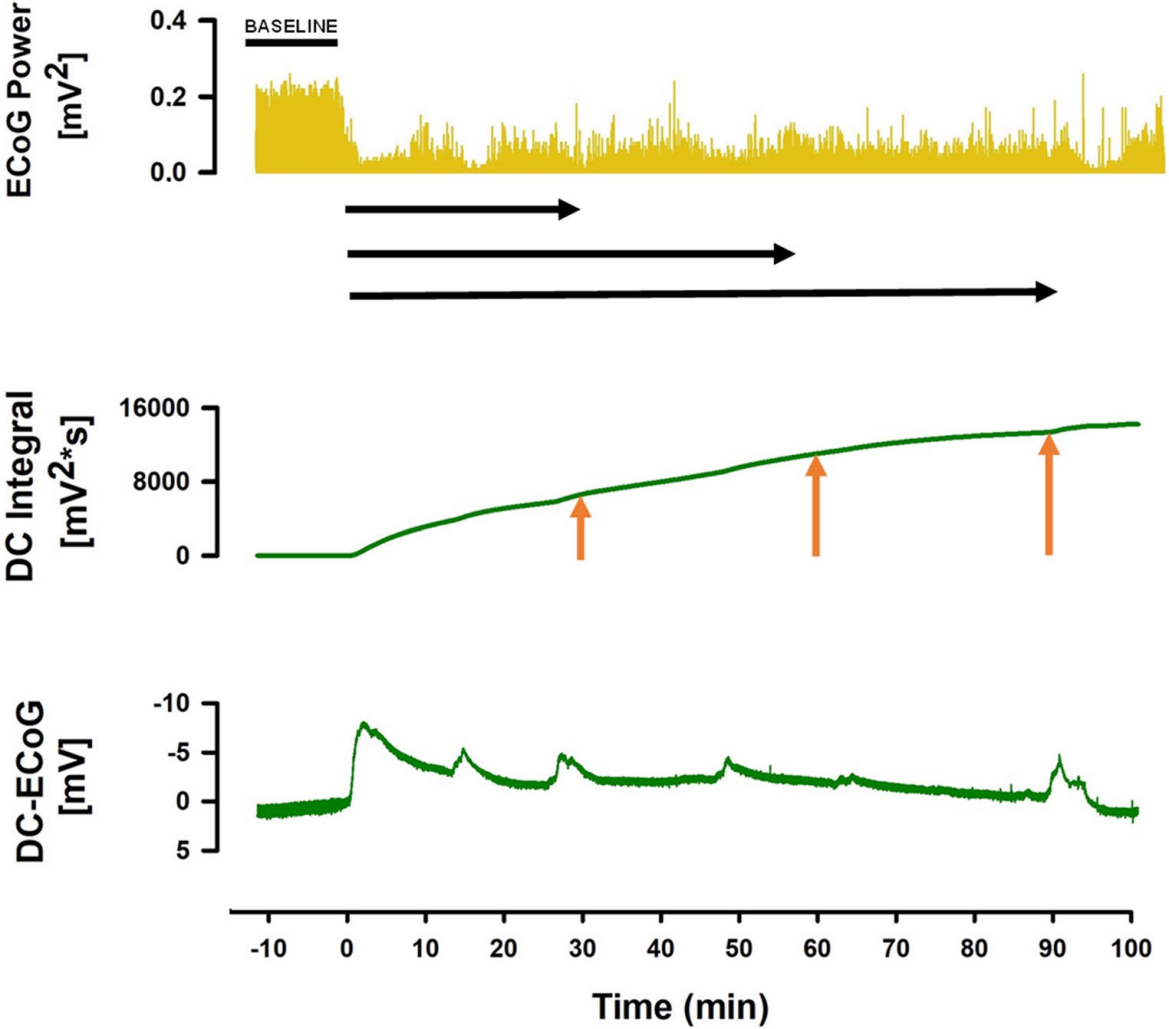
Representative traces of electrophysiological recordings (ECoG power, DC integral, and DC-ECoG). The relative changes in ECoG power (horizontal black arrows) and the integral of the DC potential (vertical red arrows) were calculated at 30, 60, and 90 min after occlusion for both the rostral (ECOG-ROST, DC-ROST) and the caudal (ECOG-CAUD, ECOG-CAUD) electrodes

The electrophysiological identification of SD followed the recommendations of the Co-Operative Studies on Brain Injury Depolarizations (COSBID) [33]. Relative changes in AC-ECoG power (bandpass: 0.5–45 Hz) and the integral of the DC potential (bandpass: 0–0.05 Hz) were calculated 30, 60, and 90 min after occlusion. When recordings showed a DC drift under baseline conditions, drift correction was performed based on a linear regression model using the statistical software package R (r-project.org). Changes in AC-ECoG power at both the rostral (ECOG-ROST) and caudal (ECOG-CAUD) electrodes were expressed relative to baseline power before MCAO. Similarly, the value of the DC-integral was determined for both the rostral (DC-ROST) and the caudal (DC-CAUD) electrode 30, 60, and 90 min after occlusion (Fig. 4).

### Statistical Analysis

Continuous variables were expressed as mean ± standard deviation or median and interquartile range (IQR) if appropriate. Discrete variables were expressed as frequencies. Based on the size of the cortical infarction, animals were artificially divided into three risk groups by using quartiles: Low-risk (infarct size below the first quartile (Q1), *n* = 7), medium-risk (infarct size within the IQR, *n* = 14) and high-risk (infarct size above the third quartile (Q3), *n* = 7).

The monotonous relationship between the continuous variables was investigated with Spearman’s rank correlation coefficient: ρ. P-values < 0.05 were regarded as statistically significant. Statistical software IBM SPSS statistics version 29.0.0. was used.

Generalization of the receiver operating characteristic (ROC) curve is proposed both for ordered and nominal three-class cases [34–36]. In our work, we used the ordered three-class ROC analysis for the three-class case: High (H), Medium (M), and Low (L). The true classification fractions were defined after selecting two ordered cut-points (CP_1_ < CP_2_): true class fraction (TCF)_H_(CP_1_), TCF_M_ (CP_1_; CP_2_) and TCF_L_(CP_2_).

The ROC surface was constructed by plotting the points (TCF_H_(CP_1_); TCF_M_ (CP_1_; CP_2_); TCF_L_(CP_2_)) for all pairs of thresholds CP_1_ < CP_2_. The volume under this three-dimensional ROC surface (VUS) is a summary index of the performance of a classification. If the diagnostic test perfectly discriminates the three classes, the ROC surface contains the point (1, 1, 1), and the value of VUS is 1. The case when TCF_H_ + TCF_M_ + TCF_L_ = 1 illustrates the uninformative classification rule, so the three classes overlap completely, and in this case the VUS is 1/6. In analogy with the two-dimension case, there are non-parametric methods analyzing the VUS [37–39]. If the diagnostic test can separate the three groups based on the VUS, finding the CP pair that separates them is important.

The generalization of the Youden index was proposed by Nakas and colleagues [40]. The Youden function is defined as the vertical distance from the ROC surface and the plane TCF_H_ + TCF_M_ + TCF_L_ = 1, the plane that represents the complete overlap of the three classes. The optimal CP pair is the one for which the maximum of J_3_(CP_1_; CP_2_) is attained:

1 $$\:{J}_{3}(C{P}_{1};\:C{P}_{2})= TCF1\left(C{P}_{1}\right) + TCF2(C{P}_{1};\:C{P}_{2})\hspace{0.17em}+\hspace{0.17em}TCF3\left(C{P}_{2}\right)$$

The analysis results in three TCFs and six False Class Fractions (FCF_HM_, FCF_HL_, FCF_MH_, FCF_ML_, FCF_LH_, FCF_LM_). ROC analysis and plots were conducted using R statistics software.

## Results

### General

All physiological parameters were within the normal range both during the surgical preparation and during MCAO. After filament occlusion of the MCA, CBF decreased. During the first 30 min of occlusion, MEAN-CBF was 38 (IQR: 29–50) % compared to the baseline value of 100% before occlusion. Upon withdrawal of the filament, CBF increased to 116 (IQR: 103–125) %.

SD-related CBF transients (*n* = 90) occurred in all rats subjected to MCAO with an average of 3.2 (IQR: 2.0–4.3) per animal. The CBF transients were mostly monophasic and hyperemic (Type III, 49%), with a smaller fraction being biphasic (Type II, 18%) and hypoemic (Type I, 25%). We were unable to classify 5 transients (5.5%) into any type because their morphology involved multiple phases (> 2) or turns (> 1). Mean, median, standard deviation and IQR of the different CBF parameters (P-AMP, N-AMP, T-AMP, P-SLOPE, N-SLOPE, All-SLOPE) at 30, 60 and 90 min are shown in Table 1.

**Table 1:** Results of the CBF and electrophysiological parameters 30, 60, and 90 min after occlusion (expressed as mean ± standard deviation (SD), median and first (Q1) and third (Q3) quartile). MEAN-CBF: relative change of CBF (%), NUM-FT: number of CBF transients, N-AMP: negative leg of the amplitude of the CBF transients (%), P-AMP: positive leg of the amplitude of the CBF transients (%), T-AMP: the sum of positive and negative peak-to-peak amplitudes of the CBF transients (%), P-SLOPE: P-AMP divided by the time until the flow reaches the positive peak (%/s), N-SLOPE: N-AMP divided by the time until the flow reaches the negative peak (%/s), ALL-SLOPES: all positive and negative slopes found within the given time interval (%/s), DC-ROST: the magnitude of the DC-Integral at the rostral electrode, DC-CAUD: the magnitude of the DC-Integral at the caudal electrode, ECOG-ROST: the relative change of the ECoG-power at the rostral electrode (%), ECOG-CAUD: the relative change of the ECoG-power at the caudal electrode (%)

		MEAN-CBF	NUM-FT	*P*-AMP	*N*-AMP	T-AMP	*P*-SLOPE
30 MIN	mean	38	1.8	11.4	12.2	13.8	0.15
	SD	16	0.8	8.0	7.7	7.3	0.11
	median	43	2.0	10.7	10.8	13.8	0.15
	Q1	29	1.0	5.4	6.4	8.6	0.07
	Q3	50	2.0	14.5	15.5	16.7	0.20
60 MIN	mean	40	2.7	12.0	12.5	14.5	0.17
	SD	16	1.4	7.2	7.7	7.5	0.10
	median	43	2.0	12.1	10.8	14.4	0.15
	Q1	30	2.0	5.9	6.7	10.6	0.08
	Q3	53	3.3	15.3	16.5	17.9	0.24
90 MIN	mean	42	3.2	12.0	13.0	14.8	0.18
	SD	17	1.6	6.60	8.1	7.7	0.10
	median	44	3.0	11.5	10.8	13.9	0.16
	Q1	31	2.0	6.60	6.7	10.6	0.10
	Q3	57	4.3	15.2	18.2	19.4	0.25
		N-SLOPE	ALL-SLOPES	DC-ROST	DC-CAUD	ECOG-ROST	ECOG-CAUD
30 MIN	mean	0.41	0.29	7677	9049	34.8	32.8
	SD	0.41	0.24	4808	5569	20.1	20.5
	median	0.26	0.19	7839	8893	34.4	31.8
	Q1	0.13	0.13	3169	4444	19.8	13.1
	Q3	0.51	0.37	10,350	13,207	45.1	45.3
60 MIN	mean	0.40	0.29	13,088	14,846	35.1	32.7
	SD	0.38	0.23	8123	10,473	20.5	21.8
	median	0.29	0.20	12,584	14,208	34.7	29.0
	Q1	0.12	0.11	4684	6513	18.5	16.1
	Q3	0.55	0.38	18,373	18,500	48.0	41.3
90 MIN	mean	0.40	0.29	15,613	18,109	38.0	35.6
	SD	0.37	0.23	9694	11,904	22.4	23.5
	median	0.29	0.19	15,393	16,443	39.7	31.3
	Q1	0.10	0.11	5419	9552	17.3	14.5
	Q3	0.59	0.43	21,144	22,825	52.7	47.9

MCAO resulted in a reduction of AC-ECoG power. The level of the AC-ECoG power relative to baseline was 38.0% (IQR: 17.3–52.7)% at the rostral (ECOG-ROST) and 35.6% (IQR: 14.5–47.9)% at the caudal (ECOG-CAUD) electrodes after 90 min of ischemia. The median of the DC integral was 15613 (IQR: 5419–21144) mV^2^*s at the rostral (DC-ROST) and 18109 (IQR: 9552–22825) mV^2^*s at the caudal (DC-CAUD) electrode after 90 min of ischemia. All electrophysiological results at different time points are presented in Table 1. Overall, the results showed that neither the CBF transients nor the AC-ECoG power changed significantly during the 90-minute ischemia, while the DC integral increased steadily.

At sacrifice after 72 h, the animals showed a striatal infarct with a median size of 37 (IQR: 19–49) mm^3^ and a cortical infarct with a median size of 67 (IQR: 14–93) mm^3^ (Fig. 1).

### Multiclass ROC Analysis

**Fig. 5:**
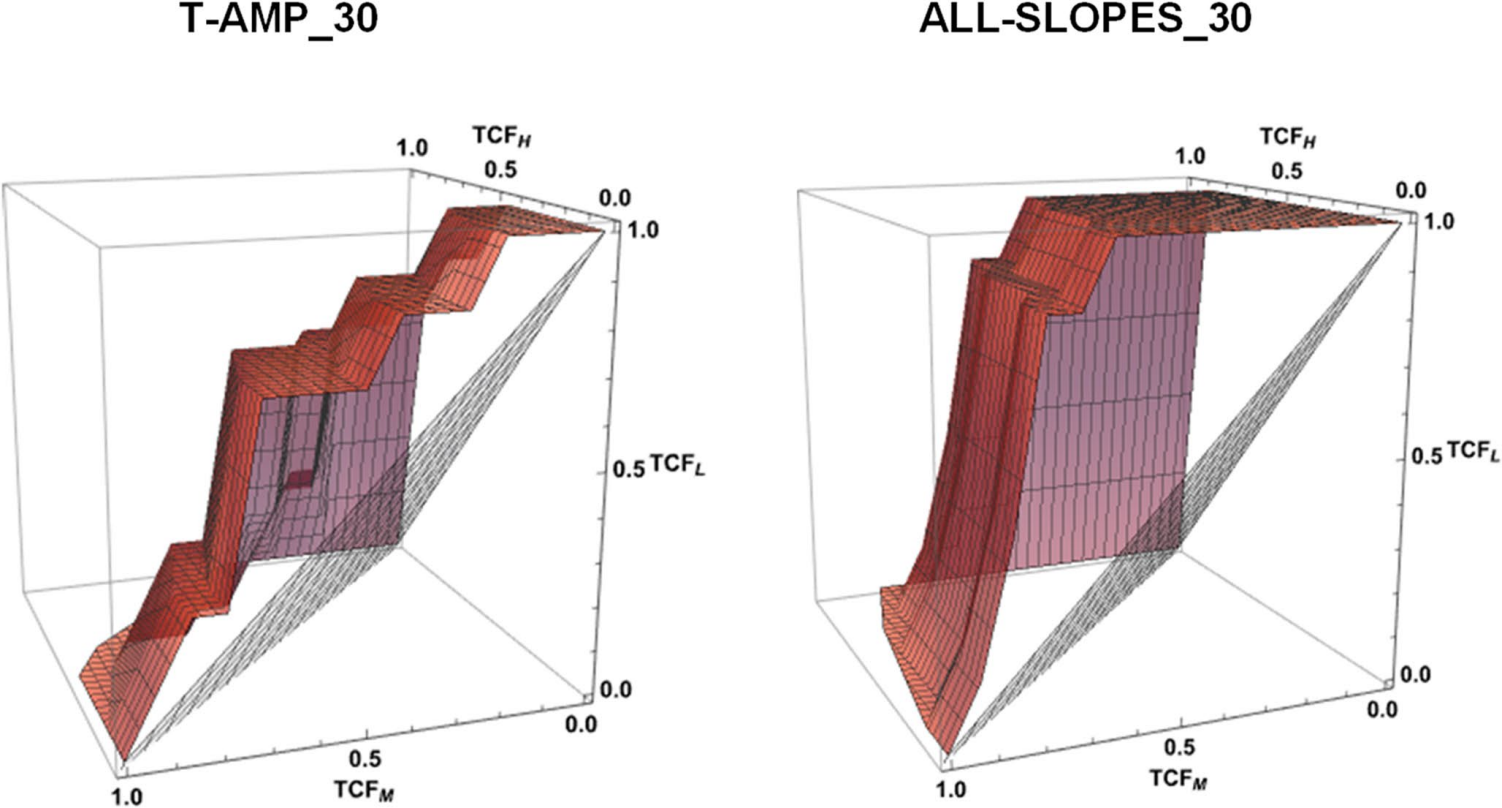
Three-dimensional ROC surface for T-AMP_30 and ALL-SLOPES_30. The true class fractions (TCF_H_, TCF_M_, TCF_L_) correspond to the optimal cut-points estimated by the Youden index

Multiclass ROC analysis revealed that parameters describing the slope of CBF transients were the best markers for distinguishing between the three risk groups (Table 2). ALL-SLOPES showed high VUS values at 30, 60, and 90 min. The relatively small sample size must be taken into account when interpreting the results. The lower limit of the confidence interval (lower bound) and the p-values are therefore particularly important. We believe that parameters with a lower bound greater than 0.3 can be suitable markers for risk stratification. Alongside ALL-SLOPES, these parameters included P-SLOPE, N-SLOPE, N-AMP, T-AMP, MEAN-CBF and DC-ROST. The three-dimensional ROC surface for ALL-SLOPES_ 30 and T-AMP_30 is shown in Fig. 5.

**Table 2:** Results of the multiclass receiver operating characteristic (ROC) analysis. The table summarizes the tested parameters based on the magnitude of the volume under the ROC surface (VUS), progressing in ascending order from bottom to top. The table also includes the confidence interval’s lower (LB) and upper bounds (UB) of the VUS and the p-values

Parameter	VUS	LB	UB	*P*-Value
P-SLOPE_90	0.85	0.60	0.95	*p* < 0.001
ALL-SLOPES_60	0.83	0.59	0.94	*p* < 0.001
N-SLOPE_90	0.83	0.58	0.94	*p* < 0.001
ALL-SLOPES_90	0.82	0.64	0.92	*p* < 0.001
ALL-SLOPES_30	0.81	0.57	0.93	*p* < 0.001
N-SLOPE_60	0.79	0.54	0.92	*p* < 0.001
N-SLOPE_30	0.76	0.48	0.91	*p* < 0.001
N-AMP_30	0.71	0.42	0.89	*p* < 0.001
P-SLOPE_60	0.71	0.41	0.90	*p* < 0.001
N-AMP_90	0.70	0.43	0.88	*p* < 0.001
P-SLOPE_30	0.66	0.37	0.87	*p* < 0.001
N-AMP_60	0.66	0.39	0.86	*p* < 0.001
DC-ROST_60	0.63	0.33	0.85	*p* < 0.001
MEAN-CBF_60	0.61	0.33	0.83	*p* < 0.001
DC-ROST_90	0.61	0.32	0.84	*p* = 0.001
MEAN-CBF_30	0.61	0.32	0.83	*p* = 0.001
DC-ROST_30	0.58	0.31	0.81	*p* = 0.001
T-AMP_30	0.55	0.30	0.77	*p* = 0.002
T-AMP_90	0.54	0.28	0.78	*p* = 0.003
NUM-FT_90	0.53	0.27	0.78	*p* = 0.006
ECOG_CAUD_90	0.53	0.28	0.76	*p* = 0.004
P-AMP_90	0.52	0.28	0.76	*p* = 0.003
DC-CAUD_30	0.51	0.25	0.75	*p* = 0.008
ECOG_CAUD_30	0.50	0.28	0.73	*p* = 0.003
T-AMP_60	0.50	0.25	0.75	*p* = 0.008
MEAN-CBF_90	0.49	0.25	0.74	*p* = 0.008
NUM-FT_60	0.49	0.26	0.73	*p* = 0.006
ECOG-CAUD_60	0.48	0.26	0.72	*p* = 0.006
DC-CAUD_60	0.46	0.23	0.71	*p* = 0.013
P-AMP_60	0.45	0.22	0.70	*p* = 0.017
NUM-FT_30	0.43	0.23	0.65	*p* = 0.011
DC-CAUD_90	0.40	0.17	0.67	*p* = 0.050
ECOG_ROST_30	0.38	0.19	0.60	*p* = 0.029
REPERFUSION	0.34	0.14	0.62	*p* = 0.087
ECOG_ROST_90	0.30	0.13	0.54	*p* = 0.114
P-AMP_30	0.28	0.10	0.57	*p* = 0.182
ECOG-ROST_60	0.26	0.11	0.50	*p* = 0.192

We also calculated the Youden-Index, the CP, and the corresponding TCF values for the best parameter (ALL-SLOPES) at three different time points (Table 3). If the goal is to differentiate the three risk groups optimally, the CP pairs should be selected to obtain the highest TCF values possible in a balanced way. For example, if the CP for ALL-SLOPES at 30 min are 0.10 (CP_1_) and 0.30 (CP_2_) then these CP distinguish the low, medium and high-risk groups with the corresponding TCF values of 0.71, 0.79 and 0.86.

**Table 3:** Presentation of the volume under the receiver operating characteristic (ROC) surface (VUS) values, the lower (CP1) and upper (CP2) cut-points as well as the corresponding true class fractions (high (TCFH)-, medium (TCFM)- and low (TCFL) risk groups) of three parameters (MEAN-CBF, T-AMP, ALL-SLOPES). True class fractions describe, for each class (high, medium and low), the proportion of observations that truly belong to that class and are correctly predicted. Suppose the experimenter’s goal is to properly separate the three risk groups, then the cut-points must be chosen from many alternatives so that the resulting TCF values for the three groups are as balanced as possible. For example, in the case of ALL-SLOPES_30, with the given pair of cut-points, the proportion of correctly classified cases is 0.79 for the medium-risk group. We obtain similar TCF values for the high-risk (0.71) and low-risk groups (0.86) as well

Parameters	VUS	CP_1_	CP_2_	TCF_H_	TCF_M_	TCF_L_
MEAN-CBF_30	0.61	16.2	44.1	0.71	0.64	0.86
MEAN-CBF_60	0.61	17.8	48.1	0.71	0.64	0.86
MEAN-CBF_90	0.49	19.5	49.1	0.71	0.57	0.71
T-AMP_30	0.55	9.5	15.9	0.71	0.5	0.71
T-AMP_60	0.50	11.1	16.3	0.71	0.5	0.71
T-AMP_90	0.54	10.6	16.9	0.71	0.57	0.71
ALL-SLOPES_30	0.81	0.10	0.30	0.71	0.79	0.86
ALL-SLOPES_60	0.83	0.09	0.29	0.71	0.79	0.86
ALL-SLOPES_90	0.82	0.10	0.40	1	0.71	0.71

If the experimental objective is only to separate the low-risk group from the rest of the animals right before the planned treatment, then it is worth carefully choosing an optimal upper CP (CP_2_). We selected CP_2_ from numerous options to achieve a high TCF_L_ value and preferably low FCF values. For example, the recommended CP_2_ for ALL-SLOPES_30 was 0.29. The TCF_L_ is satisfactorily high (86%), while the FCF between the medium and low risk group (FCF_ML_) is only 21% and between the high and low risk group (FCF_HL_) is zero (Table 4).

**Fig. 6:**
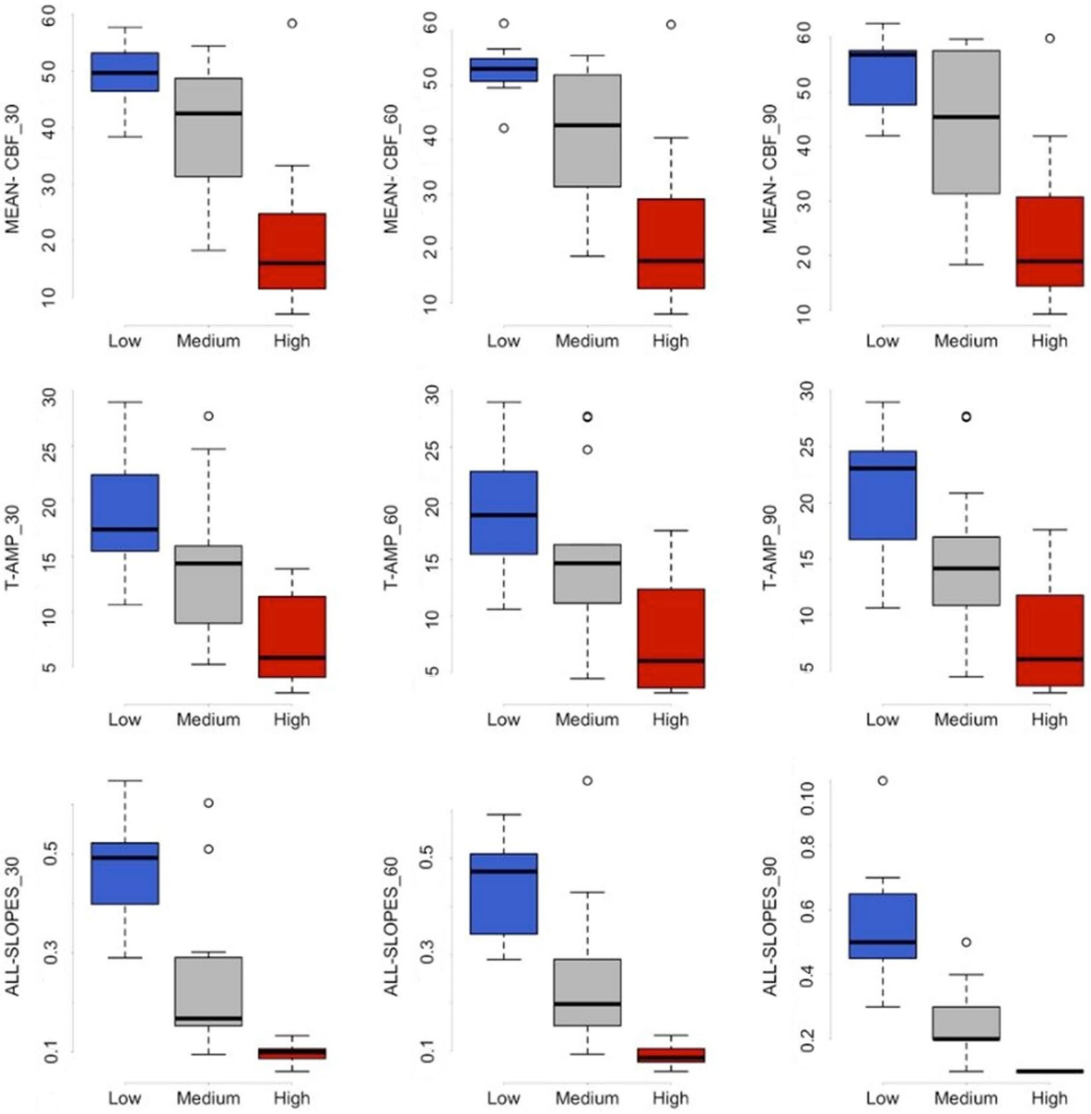
The results of descriptive statistics (boxplot analysis) of three flow parameters (MEAN-CBF, T-AMP, ALL-SLOPES) at three different time points (30, 60, 90 min). The magnitude of the MEAN-CBF and the T-AMP was calculated as a percentage (%) of the baseline flow before MCA occlusion. The measurement unit of the ALL-SLOPES is %/s. The boxplot displays the median, the quartiles, the range of values the data covers, and any outliers. The blue box indicates the low, the gray one represents the moderate, and the red one demonstrates the high-risk group

We also calculated the CP for MEAN-CBF, as it is most likely to be considered a standard parameter for risk assessment. We also included the results related to T-AMP, as the marker ALL-SLOPES is essentially derived from amplitudes. However, our results indicate that MEAN-CBF and T-AMP are clearly less effective at separating the risk groups compared to ALL-SLOPES (Fig. 6; Tables 3 and 4).

**Table 4:** Presentation of the volume under the receiver operating characteristic (ROC) surface (VUS) values, the upper cut-point (CP2) and the true class fractions for the low-risk group (TCFL) of three parameters (MEAN-CBF, T-AMP, ALL-SLOPES). The false class fractions (FCR) in relation to the high-low risk (FCFHL) and the medium-low risk (FCFML) groups are also indicated. False class fractions describe the proportion of observations not belonging to a given class. It is the “false-positive rate” for that class in multi-class ROC analysis. A low FCF means good separation. In the case of three classes, six FCFs are calculated, as follows: FCFHL, FCFML, FCFHM, FCFLM, FCFMH, FCFLH. If The experimenter’s goal is to properly separate the low-risk group, then the upper cut-point (CP2) should be chosen from among many possible options to maximize TCF while minimizing FCF simultaneously across all classes. For example, in the case of ALL-SLOPES_30, with the given CP2, the proportion of incorrectly classified cases between the low- and medium-risk groups is 0.21, while the separation between the low- and high-risk groups is perfect (zero)

Parameters	VUS	CP_2_	TCF_L_	FCF_HL_	FCF_ML_
MEAN-CBF_30	0.61	47.3	0.71	0.14	0.29
MEAN-CBF_60	0.61	50.4	0.71	0.14	0.29
MEAN-CBF_90	0.49	49.1	0.71	0.14	0.36
T-AMP_30	0.55	15.9	0.71	0.00	0.21
T-AMP_60	0.50	17.5	0.71	0.00	0.21
T-AMP_90	0.54	17.5	0.71	0.00	0.21
ALL-SLOPES_30	0.81	0.29	0.86	0.00	0.21
ALL-SLOPES_60	0.83	0.29	0.86	0.00	0.21
ALL-SLOPES_90	0.82	0.30	0.86	0.00	0.21

### Correlation Analysis

**Fig. 7:**
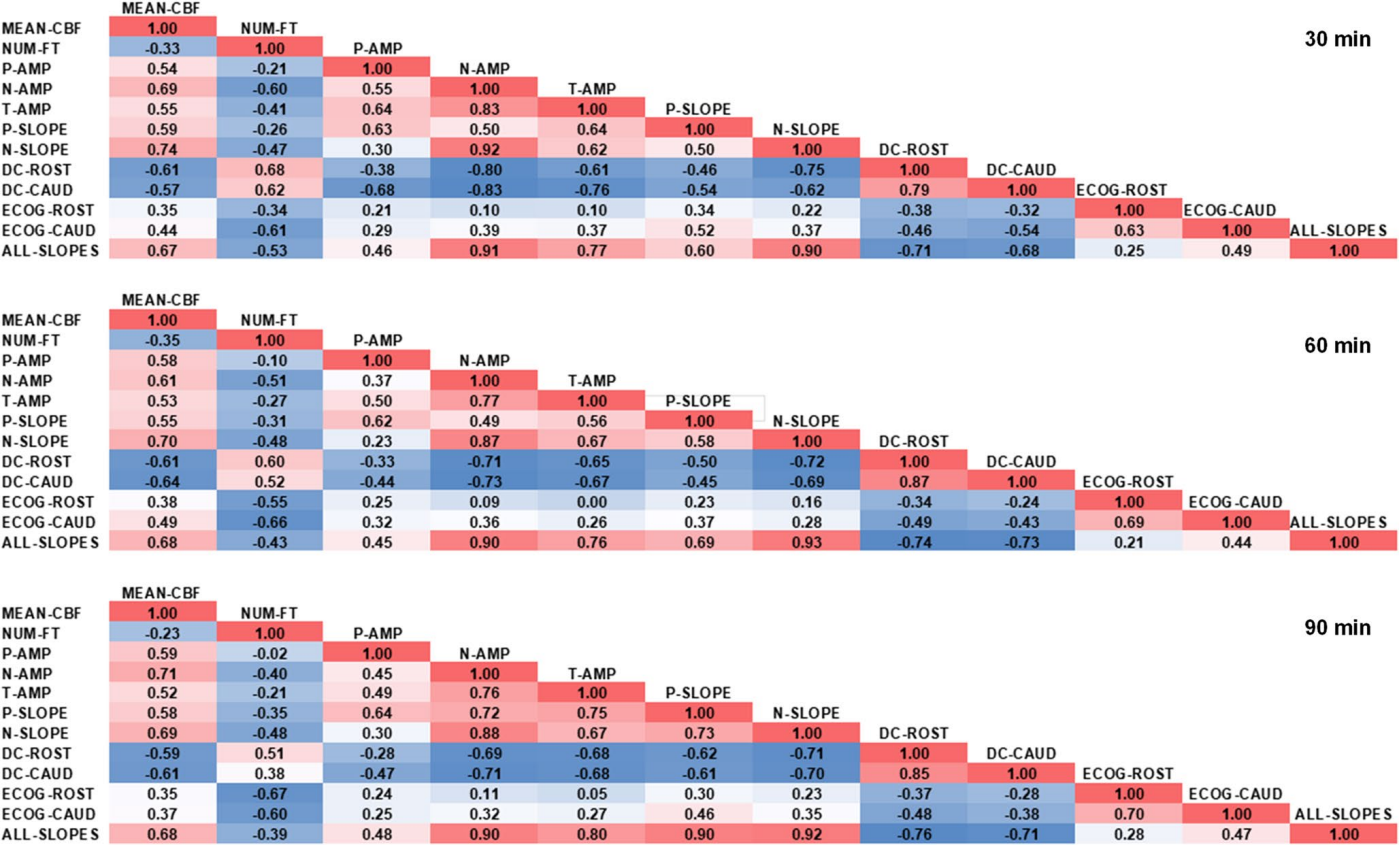
Correlograms at 30, 60 and 90 min. The cells of the tables contain the color-coded Spearman correlation values. Stronger positive values are coded with a darker red color, and stronger negative correlations are coded with a darker blue color. MEAN-CBF: relative changes of CBF (%), NUM-FT: number of flow transients, N-AMP: negative leg of the amplitude (%), P-AMP: positive leg of the amplitude (%), T-AMP: the sum of positive and negative amplitudes (peak-to-peak amplitude) (%), P-SLOPE: P-AMP divided by the time until the flow reaches the positive peak (%/s), N-SLOPE: N-AMP divided by the time until the flow reaches the negative peak, ALL-SLOPES: all positive and negative slopes found within the given time interval (%/s), DC-ROST: the magnitude of DC-integral at the rostral electrode, DC-CAUD: the magnitude of DC-integral at the caudal electrode, ECOG-ROST: the relative change of the ECoG-power at the rostral electrode (%), ECOG-CAUD: the relative change of the ECoG-power at the caudal electrode (%)

Spearman’s rank correlation revealed that the cortical infarct volume showed a good correlation - among others - with N-SLOPE (ρ= −0.84), DC-ROST (ρ = 0.73), N-AMP (ρ= −0.70), P-SLOPE (ρ= −0.69), NUM-FT (ρ = 0.67), and DC-CAUD (ρ = 0.63) 90 min after ischemia (Table 5). Interestingly, MEAN-CBF (ρ= −0.48) showed a weaker correlation with the cortical infarct volume. The p-value was < 0.01 for all correlations listed above. We also constructed correlograms 30, 60, and 90 min after ischemia based on Spearman coefficients (Fig. 7). In particular, our aim was to investigate whether there is an association between flow and electrophysiological parameters. We found that ALL-SLOPES demonstrated a negative correlation with both DC-ROST (ρ= −0.71, −0.74 and − 0.76) and DC-CAUD (ρ= −0.68, −0.73 and − 0.71) at all three time points. ECOG-ROST and ECOG-CAUD showed weaker correlations with the flow parameters, with the exception of the number of CBF transients (NUM-FT) (ρ= −0.67, ρ= −0.60) at 90 min.

**Table 5:** Spearman rank correlations of the various parameters with cortical infarct volume, sorted in descending order according to the correlation strength of the respective parameter at the end of the 90-minute ischemia

Parameter	Cortical Infarct Volume 30 min	*P*-Value	Cortical Infarct Volume 60 min	*P*-Value	Cortical Infarct Volume 90 min	*P*-Value
N-SLOPE	−0.81	*p* < 0.01	−0.81	*p* < 0.01	−0.84	*p* < 0.01
ALL-SLOPES	−0.82	*p* < 0.01	−0.81	*p* < 0.01	−0.81	*p* < 0.01
DC-ROST	0.70	*p* < 0.01	0.72	*p* < 0.01	0.73	*p* < 0.01
N-AMP	−0.76	*p* < 0.01	−0.68	*p* < 0.01	−0.70	*p* < 0.01
P-SLOPE	−0.57	*p* < 0.01	−0.57	*p* < 0.01	−0.69	*p* < 0.01
NUM-FT	0.60	*p* < 0.01	0.66	*p* < 0.01	0.67	*p* < 0.01
DC-CAUD	0.61	*p* < 0.01	0.64	*p* < 0.01	0.63	*p* < 0.01
ECOG-CAUD	−0.61	*p* < 0.01	−0.57	*p* < 0.01	−0.61	*p* < 0.01
T-AMP	−0.59	*p* < 0.01	−0.54	*p* < 0.01	−0.57	*p* < 0.01
ECOG-ROST	−0.44	*p* = 0.022	−0.41	*p* = 0.029	−0.50	*p* < 0.01
MEAN-CBF	−0.52	*p* < 0.01	−0.54	*p* < 0.01	−0.48	*p* < 0.01
P-AMP	−0.38	*p* = 0.088	−0.31	*p* = 0.166	−0.32	*p* = 0.131

## Discussion

Risk stratification enables optimal use of resources [41]. It aims to categorize a heterogeneous population of subjects into subgroups with similar involvement and treatment needs, and is frequently used in clinical stroke care [42, 43]. Segmentation can be based, for example, on patient information such as age, sex or diagnostic findings (neurological examination, neuroradiological findings, etc.). Since the neurological status of an anesthetized animal cannot be assessed, early treatment stratification can only be carried out with the help of biomarkers. An important prerequisite for a suitable biomarker would be that it should be easy to collect, i.e. minimally invasive. In a minimally invasive fashion, we here recorded CBF with LDF and DC/AC-ECoG with epidural Ag/AgCl electrodes. Our study shows that risk assessment in experimental stroke may be feasible in rats as early as 30 min after the initiation of ischemia. We have identified several parameters suitable for risk assessment, among which the slope of CBF transients to SD (ALL-SLOPES) appears to be the most promising.

### The Slope of CBF Transients

In rats and phylogenetically higher mammals, including humans, hemodynamic responses to SD show a continuum from a steep hyperemia variably followed by oligemia in metabolically intact tissue [44–46] to an increasingly ischemic response variably followed by hyperemia in tissue at risk [8, 47–49]. The mechanisms involved have been discussed previously, but are only partially understood [8, 50]. In patients, the full hemodynamic response continuum has been recorded after subarachnoid hemorrhage, malignant hemispheric stroke, and traumatic brain injury, with the strongest and longest ischemic responses to SD occurring in the context of delayed cerebral ischemia following subarachnoid hemorrhage [14, 49, 51, 52]. Similar to the spectrum observed in patients after a malignant hemispheric stroke, our rat MCAO model shows hardly any pronounced and long-lasting ischemic CBF responses to SD; rather, the observed spectrum focuses more on the transition zone from comparatively short-lasting hyperemic to hypoemic responses. Along this continuum, not only does the direction of the CBF response reverse, but the so-called slope also decreases—from metabolically intact to metabolically disturbed tissue [53], which could generally be interpreted as a decline in the responsiveness or adaptability of the system. Consistent with this, we found that ALL-SLOPES correlated positively with residual CBF (MEAN-CBF) and negatively with the DC integral (which reflects the depolarized state of brain tissue) (see Fig. 7), suggesting that the severity of ischemia and metabolic disturbance influenced the slope. Key finding of our study was that ALL-SLOPES was a relatively easy-to-determine and meaningful variable for the early assessment of the severity of ischemia and the prediction of subsequent infarct size after MCAO. Therefore, we propose that further studies be conducted to investigate the generalizability of this result. Furthermore, it would be useful to conduct further research into the complex mechanisms involved in the hemodynamic response continuum to SD, as this could potentially also provide novel therapeutic approaches.

### Time Is Brain

The effectiveness of interventions in acute ischemic stroke such as intravenous thrombolysis or endovascular treatment is time dependent [54, 55]. For example, a recent study examined the effect of mobile stroke unit (MSU) dispatch on functional outcomes in stroke patients [56]. The median value for the time from symptom onset (or last-seen-well) to dispatch was 38 min. The median dispatch-to-needle time was 50 min (IQR: 43–65) in the MSU dispatch group compared with 70 min (IQR: 59–85) in the non-MSU group. In support of the concept “time is brain”, MSU dispatch was associated with more favorable 3-month modified Rankin Scale scores after confounding adjustment (common odds ratio: 0.82 (95% confidence interval: 0.71–0.94). Generally, it is assumed that interventions are most effective when the time between the onset of symptoms and treatment is less than 60 min. This period is therefore referred to as the “golden hour” [57]. However, it is estimated that the maximum size of the lesion in diffusion-weighted imaging (DWI) is only reached after about 70 h in patients [58]. In line with this, Kowoll et al. found that in 21 of 44 patients (48%), who underwent an initial MRI scan 57 ± 41 h after the onset of MCAO causing MHS, a second MRI scan 139 ± 28 h after the onset of MCAO still showed > 5% infarct progression [17]. After MCAO in non-human primates, it has been reported that the maximum lesion volume was also reached later than 48 h [59]. In contrast, it has been suggested that infarction growth stops within 3 to 6 h of permanent MCAO in rodents [60]. An important modifier of the time course is reperfusion, as energy metabolism typically recovers depending on the timing of reperfusion, thereby restoring energy availability to activate membrane pumps and allowing the tissue in the core area of ischemia to recover from SD. If transient ischemia and SD in the core area lasted only about 15 min, complete recovery occurred in rats after transient MCAO [14]. However, if the point of no return, the so called commitment point, has already been exceeded, secondary delayed cell death typically develops after reperfusion, occurring in rodents in the area of primary energy failure within 6 to 12 h after the onset of ischemia [61]. In total, estimates suggest that infarct growth takes about 24 h after transient MCAO [19]. Overall, transient ischemia therefore reduces and delays infarct growth. However, this does not change the fact that the evolution of neuronal cell death in rodents is significantly faster than in primates, which is consistent with a large amount of data showing that the metabolism of rodents is considerably faster than that of primates [62]. For example, m/tRNA turnover in rats is about 2.5 times faster than in humans, while protein turnover is about 10 times faster. Basal metabolic rate in rats is about 6.4 times faster. Heart rate is on average about 4.7 times faster and respiratory rate about 6.3 times faster, to name just a few examples. Based on current knowledge, the damage equivalent that occurs in humans during the “golden hour” is therefore achieved in rats in a significantly shorter ischemia time than one hour. However, even in Sprague-Dawley rats, a significant diffusion/perfusion mismatch can still be observed 60 min after the onset of ischemia [60]. Overall, taking into account the logistical developments in preclinical care of stroke patients and the species differences outlined above, we assume that the early time windows of 30 and 60 min chosen in our rat model are particularly relevant for the development of neuroprotection strategies that could be translatable to clinical settings. If the availability of monitoring equipment is a factor, a 30-minute interval may be the best compromise between cost and benefit for monitoring during a 90-minute MCAO.

### Parameters Suitable for Early Risk Assessment

Based on the results of the multiclass ROC analysis, we consider the following parameters as potential biomarkers for risk assessment at almost all selected time points: ALL-SLOPES, N-SLOPE, P-SLOPE, N-AMP, DC-ROST, T-AMP, and MEAN-CBF. The lower bound of the confidence interval (> 0.3) and the p-value (≤ 0.001) set these parameters apart from the others. We primarily selected ALL-SLOPES for further analysis because it has a large sample size (each CBF transient has positive or negative slopes), and overall, it was the best marker at 30, 60, and 90 min based on the VUS.

During the multiclass ROC analysis, we generated numerous CP and the corresponding TCF/FCF values for ALL-SLOPES. For example, using CP of 0.10 and 0.30 at 30 min, we obtained TCF values that can effectively and in a balanced manner distinguish between the three risk groups (Table 4). If the goal is only to separate the low-risk group from the medium-risk group at 30 min, it can be effectively achieved with a CP of 0.29 (Table 4).

The slope of the CBF transients has already been previously mentioned as a promising biomarker. Thus, a correlation between positive slope and pre-SD level of CBF was found in rats subjected to MCAO [53]. Similarly, associations between local risk of infarction and positive slopes of CBF transients as well as oxy- and deoxyhemoglobin transients were recently demonstrated in a rat study of distal MCAO [63]. In patients during neurocritical care after ischemic stroke, responses of tissue partial pressure of oxygen (p_ti_O_2_) to SD are more readily available than CBF responses or responses of oxy- and deoxyhemoglobin. The relationship between CBF and p_ti_O_2_ responses is complex, as the direction of the responses can be equal or opposite [64–66]. However, analogous to our results in rats, stronger p_ti_O_2_ responses to SD in patients have recently been found to be an indicator of a more favorable clinical prognosis after MHS due to MCAO [67].

We have also calculated CP values and TCF/FCF values for MEAN-CBF, as it can be considered a standard parameter for risk assessment [68, 69]. In addition, we performed such analyses for T-AMP, since the best parameter (ALL-SLOPES) is essentially derived from the amplitudes and T-AMP is easy to measure during the experiment. Although both parameters seemed promising based on VUS and p-values, they appeared less so after calculating TCF/FCF (Table 3). T-AMP is suitable to some degree for distinguishing the low-risk group (Table 4).

### The Slope of CBF Transients in Light of the STAIR Recommendations

The STAIR 1999 recommendations advise that experimenters monitor CBF in rats undergoing MCAO using LDF to ensure a ≥ 60% drop in CBF during filament occlusion (i.e., residual CBF ≤ 40% of baseline) [68]. Based on the results of the present study we recommend revisiting the recommendations of the STAIR guideline for the following reasons: There is a lack of information on the experimental conditions under which the CBF threshold was determined, and it is known that the species, type of anesthesia, age of the animals, duration of ischemia, position of the LDF probe, etc. influence variability [69]. In addition, factors that have not been sufficiently examined so far may also affect variability, such as the method of anesthesia administration (e.g., nose cone vs. mechanical ventilation), the position of the animals (prone versus supine), the experimenter’s experience, or the surgical technique. In J.L.‘s personal experience, discrepancies can even occur when the same experimenter conducts the same experiments in different laboratories. Therefore, we suggest that while general guidelines can serve as a point of orientation, each laboratory should ultimately set its own CBF limits. More importantly, however, MEAN-CBF did not appear to be the most effective biomarker for early risk stratification in the present study. Other CBF-derived markers (e.g., ALL-SLOPES) but also electrophysiological markers such as DC-ROST performed better. Therefore, it could even be interesting to investigate in a multi-center study whether a parameter such as ALL-SLOPES would be more robust for a general guideline than the simple drop in CBF.

### Electrophysiological Parameters

It is known from experimental and clinical studies that the significance of electrophysiological measurements decreases drastically from the subdural recording site to the epidural recording site to the scalp [70, 71]. In other words, whilst epidural recordings are less invasive than subdural recordings, they reflect intracortical changes to only a limited extent in both humans and animals. In line with this, our epidurally measured electrophysiological biomarkers proved to be less promising for risk assessment than the CBF parameters. Although the implantation of epidural electrodes involves only minimal additional effort, it is advantageous overall that the CBF parameters allowed for the best prediction, as the positioning of the LDF probe requires even less effort than the electrophysiological measurements. The positioning of an LDF probe is recommended anyway for quality control in MCAO experiments on rats in order to detect ischemia and, in the case of transient MCAO, also reperfusion [26, 68, 69]. Based on VUS values and p-values, DC-ROST was the best electrophysiological marker. ALL-SLOPES and DC-ROST showed a negative correlation. In agreement with this result, Feuerstein et al. observed a negative correlation between the positive slope of the CBF transients and the DC durations of SD, while there was a positive correlation between the positive slope and the EEG power [53]. Feuerstein et al. also reported CBF transients to spontaneous SD using a subdural optoelectrode strip in one patient with subarachnoid hemorrhage and one patient with traumatic brain injury. In line with the MCAO animal model, they found a negative correlation between the positive slope of the CBF transients and the duration of ECoG depression.

### Potential Statistical Impact

To illustrate the potential impact, we randomly assigned the experimental animals from the current study into two hypothetical groups (*n* = 14 + 14), mimicking an interventional design (treated versus non-treated). In the first scenario, low-risk animals were allocated to the treated and non-treated groups in a 4:3 ratio. In the second scenario, 5 low-risk animals were assigned to the treated group and 2 to the non-treated group. The dependent variable was the cortical infarct size. When all animals were included (*n* = 14 + 14) the standard deviation of the within-group differences was 46.3. In scenario 1, the effective group sizes were reduced to n′=10 + 11, and the standard deviation decreased to 35.3, corresponding to a 24% reduction in variability relevant for an independent-samples t-test. In scenario 2, the effective sample sizes were n″=9 + 12, and the standard deviation further decreased to 34.9, representing a 24.5% reduction. These findings indicate that identifying and excluding low-risk animals could yield an approximate 24% reduction in standard deviation for analyses based on an independent-samples t-test.

### Limitations of the Study

We included 28 animals in the original experiment based on the preliminary statistical estimate. However, this number of animals is relatively low for the multiclass ROC analysis, so the results should be interpreted with caution. For example, in the case of VUS values, we considered only the lower bound (LB) values. It would also be interesting to combine various parameters with each other; however, due to the relatively low number of animals, multivariate analysis is not possible.

Additionally, the present experimental setup is unique to a certain degree. For example, we placed the LDF probe at a prominent point in the MCA supply area, but the position of the LDF probe may influence the final result [69]. The experiments were conducted on young adult Sprague-Dawley rats under isoflurane anesthesia in a transient (90 min) ischemia model. The strain, the age of animals, the anesthesia, and the duration of the ischemia can all influence the results [28, 60, 72, 73]. CP values may also vary from laboratory to laboratory. In practice, we would therefore recommend that a given laboratory determines its own CP values for ALL-SLOPES. Another limitation of the study is that the CP values for risk-stratification were not validated using an independent dataset. Furthermore, the current sample size was too small to allow for a reliable division into separate training and test sets.

We would like to add that it is probably not trivial to transfer our approach to mice. In general, it is more difficult with mice than with rats to position a device such as a laser-Doppler probe in such a way that continuous recording without interruption is possible throughout the pre-ischemia phase, the ischemia phase, and the reperfusion phase. This is possible, for example, with a straight microtip probe (e.g., MT B500-0L240, Perimed, Järfälla, Sweden) [74]. However, the recording quality with such a probe is not comparable to that of a conventional LDF probe. Moreover, mice are not only more delicate than rats, but also less stable. Therefore, mice are usually allowed to wake up and move during the ischemia phase. Anesthesia is then reintroduced before the monofilament is removed from the MCA for reperfusion [75]. The recording quality is naturally further impaired during such a waking phase by motion artifacts.

Most importantly, however, the hemodynamic response continuum to SD in mice differs from that in higher mammals [76]. Thus, the mouse response in normal tissue starts with pronounced initial hypoperfusion instead of hyperemia, followed by a short peak that barely reaches baseline and renewed, very prolonged CBF reduction by ~ 60% [77]. Nevertheless, it would be useful to examine whether rules derived from the method proposed in the present article could also be suitable for predicting infarcts in mice.

## Conclusion

Risk stratification using biomarkers could be applicable in many experimental stroke settings, particularly in early-phase neuroprotection studies. Based on our experience, perhaps the most significant advantage of risk assessment is the ability to separate the low-risk group from the other animals, as the efficacy of the neuroprotective agent cannot be determined in animals where the size of the cortical infarct would be zero or very small even without treatment. The random and uneven distribution of low-risk animals in treated and untreated groups easily distorts the statistical analysis. By identifying the low-risk group, the experimenter can also exclude these animals early from the experiment, sparing them from further suffering. This is particularly relevant because animals usually wake up again after MCAO and, for example, in our study were not sacrificed until 72 h following MCAO. Accordingly, our suggestion of excluding the low-risk group from neuroprotection studies at an early stage and euthanizing them without reawakening them is in line with the principle of refinement in animal welfare [24]. In contrast, we recommend not excluding the high-risk group for particularly large infarcts, but rather treating them as a separate group during randomization in order to be able to determine the outcome for animals in the medium-risk and high-risk groups separately. This can be helpful, for example, in order to perform a meaningful subgroup analysis later on.

## Data Availability

The data are available from the first author upon reasonable request.

## Abbreviations

AC: alternating current
ALL-SLOPES: all the slopes of cerebral blood flow transients
CAUD: caudal
CBF: cerebral blood flow
COSBID: Co-Operative Studies on Brain Injury Depolarizations
CP: cut-point
DC: direct current
ECoG: electrocorticography
FCF: false class fraction
LDF: laser-Doppler Flowmetry
MCA: middle cerebral artery
MEAN-CBF: the mean of cerebral blood flow after occlusion
MSU: Mobile Stroke Unit
N-AMP: negative leg of the amplitude of cerebral blood flow transients
N-SLOPE: negative slope of cerebral blood flow transients
NUM-FT: number of cerebral blood flow transients
P-AMP: positive leg of the amplitude of cerebral blood flow transients
P-SLOPE: positive slope of cerebral blood flow transients
T-AMP: total (peak-to-peak) amplitude of cerebral blood flow transients
ROC: receiver operating characteristic
ROST: rostral
SD: spreading depolarization
TCF: true class fraction
VUS: volume under the receiver operating characteristic surface
